# Infant Salmonella enterica Meningitis: A Rare Case Report and Review of Literature

**DOI:** 10.7759/cureus.55405

**Published:** 2024-03-02

**Authors:** Khalid N Ali, Farman O Shareef, Jeza M Abdul Aziz, Zana B Najmadden, Ari H Karim

**Affiliations:** 1 Biomedical Sciences, Komar University of Science and Technology, Sulaymaniyah, IRQ; 2 Medical Laboratory Science, Charmo University, Sulaymaniyah, IRQ; 3 Baxshin Research Center, Baxshin Hospital, Sulaymaniyah, IRQ; 4 Research Center, University of Halabja, Halabja, IRQ; 5 Baxshin Research center, Baxshin Hospital, Sulaymaniyah, IRQ; 6 Nursing, Azmar Technical and Vocational Institute, Sulaymaniyah, IRQ

**Keywords:** infant, antibiotic susceptibility testing, cerebrospinal fluid culture, meningitis, salmonella

## Abstract

Meningitis caused by *Salmonella enterica* can be a fatal condition that is more common in low- and middle-income countries and uncommon in infants. This case of a 2-month-old male infant reported *Salmonella* meningitis symptoms, such as fever, irritability, altered sensorium, and diarrhoea. Clinical examination revealed bulging anterior fontanelles, dehydration, and sunken eyes. Screening for normal hearing, cranial ultrasound, and magnetic resonance imaging (MRI) revealed no brain abnormalities. A cerebrospinal fluid (CSF) culture revealed gram-negative *Salmonella enterica* bacilli. Treatment with meropenem and ampicillin was initiated after antibiotic susceptibility testing showed sensitivity. The patient’s cerebrospinal fluid parameters and bacterial growth improved after antibiotic therapy. Two weeks later, the baby was neurologically healthy and discharged. Paediatricians should be aware that *Salmonella enterica* can cause meningitis in children with non-specific symptoms.

## Introduction

*Salmonella* are members of the Enterobacteriaceae family and are gram-negative bacilli (2-5 µm X 0.5-1.5 µm), facultative anaerobic bacteria. *Salmonella *is motile with a peritrichous flagellar pattern. In the Enterobacteriaceae family, *Salmonella* forms a complex group of bacteria comprising two species, six subspecies, and several serovars [1]. The two species currently recognised in the genera are *Salmonella enterica* and *Salmonella bongori*, which are medically valuable pathogens that infect both humans and animals [1]. The main host organ of *Salmonella serovars* is the gastrointestinal tract of humans, farm animals, and wild animals [2]. After reaching the gastrointestinal tract, *Salmonella* colonises the area and bacterial cells are excreted in diarrhoeal faeces, from which insects and other animals may transmit bacterial cells to different vulnerable places and foods [3]. Besides the gastrointestinal tract, other organs, such as the blood and brain, may also suffer from salmonellosis, causing bacteraemia and meningitis [4].

Acute meningitis caused by *Salmonella* species is a rare clinical observation that manifests significantly in newborns and young infants and is associated with a mortality rate of approximately 90% [5]. *Salmonella* meningitis usually manifests with nonspecific symptoms, including fever, irritability, lethargy, and an altered level of consciousness, making its diagnosis difficult. Organisms can be isolated from either cerebrospinal fluid (CSF) or blood cultures as an essential step in identification [6]. We present the case of a 2-month-old male infant who presented with signs and symptoms of meningitis.

## Case presentation

A 2-month male infant (birth weight: 5 kg and living in a rural area) born via vaginal delivery was admitted to the hospital with fever, poor feeding, irritability, diarrhoea, altered sensorium, lactose intolerance. On physical examination, the infant had a bulging anterior fontanelle, dehydration, dry eyes, little to no tears when crying or sunken, a clear chest, and a soft abdomen. Neurological examination revealed left-sided facial palsy with a normal fundus and pupillary size. Oxygen saturation was 85% without oxygen, respiratory rate was 50 cycles/min, blood pressure was 55/45 mmHg, and body temperature was ≥39°C. Neonatal hearing screening tests, including bilateral normal middle ear pressure (Type A) tympanogram, bilateral otoacoustic emission test, and bilateral auditory brainstem test results, were normal. Ultrasonography of the cranium and MRI of the head revealed a normal brain. C-reactive protein (CRP) was at 225.51 mg/L and CSF was colorless and semi-turbid. Biochemical analysis of the CSF indicated CSF protein at 4614 mg/L, CSF glucose at 1.0 mg/dL, WBC-CSF at 144cells/µL, RBC-CSF at 1.0 ×10^3^/µL (Table 1), polymorphonuclear leukocyte (PMN) at 86 cells, and total cell count-CSF at 147 cells. The culture results showed heavy growth of gram-negative bacilli. Identification and susceptibility to antibiotics were based on an automated Vitek-2 instrument (bioMérieux, Marcyl’Etoile, France). The results revealed *Salmonella enterica* that was sensitive to ampicillin (≤2µg/ml), piperacillin (≤2µg/ml), trimethoprim-sulfamethoxazole (≤20µ/ml), tigecycline (0.25 µg/ml), ciprofloxacin (≤0.25µ/ml), meropenem (≤0.25µ/ml), ertapenem (≤0.12µ/ml), cefepime (≤0.12µ/ml), and ceftazidime (0.25µ/ml). The patient was admitted to the intensive care unit (ICU) for one week. Before culture, empirical treatment was initiated with intravenous vancomycin 60 mg/kg four times per day and cefotaxime 100 mg/kg four times per day. Culture results showed that the infant was infected with *Salmonella enterica*. The patient was treated with 10 mg/kg of meropenem three times per day and ampicillin 200 mg/kg four times per day intravenously for 14 days. After seven days of antibiotic treatment, the repeat CSF analysis showed a decrease in WBC (140 cells/µL), RBC (769 cells/µL), and protein (148 mg/dl); however, CSF glucose increased to 32 mg/dL. The infant continued treatment for seven days. The third CSF analysis indicated that all biochemical tests were normal, and the culture results showed no bacterial growth (Table 1).

**Table 1:** Characteristics of cerebrospinal fluid specimens

Laboratory test	1 week	2 week	3 week	Normal values
WBC	144cells/µl	140 cells/µl	4 cells/µl	0-5 cells/µl
RBC	1.0 ×10^3^/µl	760	4	0-5 cells/µl
Protein	461.4 mg/dl	148	43	15-45 mg/dl
glucose	1.0 mg/dl	32	60	45-80 mg/dl
appearance	Semi turbid	Turbid	clear	clear
Gram stain	Negative, bacilli shape	Not done	No present bacteria	
Culture	Salmonella enterica	Not done	No growth	

The patient was discharged from the hospital after two weeks. The infant was observed during the follow-up visit two weeks after discharge. The neurological examination results were normal.

## Discussion

*Salmonella enterica* serovars are the leading cause of invasive systemic and local infections, including meningitis, in humans. They cause high morbidity and fatality [7], especially in children and immunocompromised adults [8]. Because of the consequences of this infection, early diagnosis and treatment are of paramount importance. To date, meningitis due to *Salmonella enterica* has not been widely reported, and only a few cases have been documented. *Salmonella* meningitis accounts for 0.8-6% of bacterial meningitis cases, and the majority of affected patients are infants [9] and, more specifically, neonates, because of reduced gastric acidity and peristalsis [10]. This study highlights previously undescribed bacterial and clinical features of invasive infections in infants in Iraq. Carneiro et al. reported the first case of *Salmonella enterica* meningitis in a four-month-old infant in Brazil. Here, the patient was a 4-month-old infant, presenting with symptoms of fever, diarrhoea, irritability, and altered level of consciousness. Subsequent investigations, including CSF culture, revealed gram-negative bacilli that were sensitive to most antibiotics, including ampicillin, ceftriaxone, ceftazidime, chloramphenicol, and trimethoprim. Fortunately, the patient survived [11]. Another interesting study by Lakew et al. described a 13-day-old neonate with* Salmonella enterica* meningitis; the patient had no remarkable prenatal, natal, or postnatal events. CSF culture and sensitivity tests revealed gram-negative rods that were susceptible to cefotaxime, chloramphenicol, ceftriaxone, and ciprofloxacin. Despite an appropriate management plan, the patient did not respond to medication and deteriorated further. Complications of the disease, such as frequent focal convulsions and hydrocephalus, started, and 13 days later, the patient died [12]. A case series study by Leonard et al. concluded that infection is more likely to occur in infants with concomitant HIV infection by 70% [13]. Interestingly, the patient in the current study had no malnutrition, malaria, or immunocompromised status, in contrast to what was mentioned in other studies [8] [14]. 

The source of the infection is usually food, often linked to animal reservoirs, and sometimes nosocomial infection is documented in neonatal wards [15]. However, in the current study, direct food at birth was unlikely because the infant only drinks milk, and the source may be from another family member, contaminated bottles, pacifiers, or clothes. The infant in this study shared common clinical features with the case reported in Brazil, such as fever, loose bowel motion, decreased oral intake, and altered sensorium level. Importantly, the documented strain was sensitive to most antibiotics, such as ampicillin, piperacillin, trimethoprim-sulfamethoxazole, tigecycline, ciprofloxacin, meropenem, ertapenem, cefepime, and ceftazidime. This was similar to what was reported by Carneiro et al. [11] and Karim et al. [9], but in contrast with the study by Vaagland et al. [16] (in which the reported strain was resistant to first-line antibiotics, including ampicillin and gentamicin), and that reported by Mohan et al., which was resistant to ampicillin 21%, tetracycline 61%, trimethoprim 5%, and chloramphenicol 21% [7]. Regarding the CSF parameters of our case, pleocytosis, increased protein, and decreased sugar were observed, but the differential cells of the CSF were mostly neutrophils, similar to that reported by Carneiro et al. [11], Vaagland et al, and Karim et al., but in contrast to what was reported by Lakew et al. where most of the CSF leukocytes were lymphocytes [12]. The American Academy of Paediatrics (AAP) recommends that *Salmonella* meningitis should receive treatment for four weeks or three weeks after sterilisation of CSF [17], even if there is a response to medications owing to the chance of relapse. Pavlova et al. reported a 3-month-old infant with *Salmonella enterica *meningoencephalitis; she got right-sided weakness and dilated subarachnoid spaces. She developed a recurrence of meningitis two weeks after the first discharge, after which she developed bilateral subdural effusions. After an initial improvement in the hospital, she passed away one week after the second discharge [18]. Notably, all patients affected by this pathogen should undergo repeat CSF examination because there is a chance of persistence of the bacteria in the CSF, which is linked to a high minimum inhibitory concentration (MIC) of antibiotics and the risk of antibiotic resistance [19]. Antibiotic resistance is a growing global health concern, and this microorganism is currently resistant to some antibiotics, including ampicillin, cephalosporins, and quinolones [9]. Fortunately, the current patient was sensitive to these antibiotics.

## Conclusions

Paediatricians should be aware of the potential for meningitis caused by *Salmonella enterica* in children, as this infection can have non-specific clinical symptoms. Prompt identification of meningitis symptoms with appropriate diagnostic investigations and the accurate modification of antibiotic medication based on culture sensitivity data played a vital role in achieving a positive outcome. 
